# Monitoring Plant Status and Fertilization Strategy through Multispectral Images

**DOI:** 10.3390/s20020435

**Published:** 2020-01-13

**Authors:** Matheus Cardim Ferreira Lima, Anne Krus, Constantino Valero, Antonio Barrientos, Jaime del Cerro, Juan Jesús Roldán-Gómez

**Affiliations:** 1 Department of Agroforest Ecosystems, ETSI Agrónomos, Universidad Politécnica de Valencia, 46022 Valencia, Spain; 2 Research and Extension Unit (AGDR), Food and Agriculture Organization of the United Nations (FAO), 00153 Rome, Italy; 3 Department of Agroforest Engineering, ETSI Agronómica, Alimentaria y de Biosistemas, Universidad Politécnica de Madrid, 28040 Madrid, Spain; a.m.krus@upm.es (A.K.); constantino.valero@upm.es (C.V.); 4 Centre for Automation and Robotics (CSIC-UPM), 28006 Madrid, Spain; antonio.barrientos@upm.es (A.B.); j.cerro@upm.es (J.d.C.); or juan.roldan@uam.es (J.J.R.-G.); 5 Department of Computer Engineering, Higher Polytechnic School, Autonomous University of Madrid (UAM), 28049 Madrid, Spain

**Keywords:** multispectral image, computer vision, precision agriculture, vegetation indices, morphological features

## Abstract

A crop monitoring system was developed for the supervision of organic fertilization status on tomato plants at early stages. An automatic and nondestructive approach was used to analyze tomato plants with different levels of water-soluble organic fertilizer (3 + 5 NK) and vermicompost. The evaluation system was composed by a multispectral camera with five lenses: green (550 nm), red (660 nm), red edge (735 nm), near infrared (790 nm), RGB, and a computational image processing system. The water-soluble fertilizer was applied weekly in four different treatments: (T0: 0 mL, T1: 6.25 mL, T2: 12.5 mL and T3: 25 mL) and the vermicomposting was added in Weeks 1 and 5. The trial was conducted in a greenhouse and 192 images were taken with each lens. A plant segmentation algorithm was developed and several vegetation indices were calculated. On top of calculating indices, multiple morphological features were obtained through image processing techniques. The morphological features were revealed to be more feasible to distinguish between the control and the organic fertilized plants than the vegetation indices. The system was developed in order to be assembled in a precision organic fertilization robotic platform.

## 1. Introduction

Environmental protection allied with health concerns represents an increasingly important trend in the consumer behaviour and has led to the development of green products and organic markets [1]. These markets have experienced exponential growth in recent years, especially in the organic markets that address multiple consumer concerns relating to health, food safety and environmental conservation [2]. Tomatoes are one of the vegetable crops with a higher demand for fertilization, with recommended doses close to 400 kg∙ha^−1^ depending on soil type [3].

Deficient uses of organic fertilization can cause “abiotic diseases” on plants, decrease the suppressive effect of the soil, and the readiness of plants to defend themselves against attacks of plant pathogens [4]. Nutrient deficiency affects the plant growth, with consequences in the production and the profitability of the organic field. On the other hand, the indiscriminate use of manure and soluble fertilizers prolong the vegetative state of the plant with the abundance of young tissue, making the plants more susceptible to plant pathogen attacks for a longer time [4,5]. Further, a considerable amount of the fertilizers cannot be totally absorbed by the plants. These compounds can run off into waterways, leached into groundwater, or become lost in gaseous form. The leachate liquid derived from the organic fertilizer can cause pollution of groundwater producing toxic algae blooms, accelerating eutrophication, and reducing biodiversity [6].

Meta-analysis studies indicate that the optimization of the use of nitrogen fertilization in tomatoes can decrease costs and environmental impact maintaining the same yield levels [7]. Focusing on that, the European commission fomented projects to study cost-efficiency technologies and bring innovations to reduce the dependency of contentious inputs in the organic production systems.

The SUREVEG (SUREVEG stands for “Strip-cropping and recycling of waste for biodiverse and resource-efficient intensive vegetable production” and is funded via ERANET Core Organic Cofund. More info: https://projects.au.dk/coreorganiccofund/core-organic-cofund-projects/sureveg/) Project seeks to develop and implement innovations for intensive cropping systems using strip-cropping and fertility strategies. These farming systems consists of inter-row cropping with different families and species in the same plot. The increase in biodiversity enhances the resilience of the field against soil-borne disease, and pest attacks, which at the same time increase the soil protection against erosion and nutrient depletion.

One of the research lines aims to develop and assess smart technologies for the management of strip-cropping systems. These smart technologies are focused on the use of precise fertilization methods to reduce the dependency on biopesticides and non-organic fertilizers, improve soil fertility in intensive vegetable cropping systems and therefore bring a positive impact on water quality and landscape biodiversity.

The field of precision fertilization aims to optimize the use of resources in time and space. Smart technologies were developed for monitoring the nutrient status of plants and control variable rate applications in broad, monocrop, and conventional fields [8].

Initially these technologies were based on soil samples, yield mapping, and automatic guidance [9]. After that, the advances in sensor technology-enabled nondestructive optical approaches and the use of satellite and unmanned aerial vehicles images were added to the system [10]. Nowadays new embedded devices have been implemented in order to analyze the nutrient status of the crop in real-time using high-resolution image sensors at plant level [11].

These sensors are based on multispectral images and use vegetation indices to obtain the best correlation with the nutrient status of the arable crops [12,13,14].

In order to optimize the use of the spectral information acquired by the multispectral camera for monitoring the nutrient content of plants, a principal component analysis approach can be used. This methodology uses orthogonal transformations to convert the spectral measurements at different wavelengths into an orthogonal system of eigenvectors. This approach combined with multiple linear regression allows to create new vegetation indices, reconstruct the leaf reflectance spectra, and predict the leaf biochemical contents with high accuracy [15].

These sensors are also being used for many applications regarding plant analysis. The multispectral images can be used for counting plants in orchard fields [16], estimate biomass, productivity, canopy traits [17], phenological state [18] and mortality of forest trees [19], and can also estimate the damage level of insect pests in forest and crops [20], assess hydric deficiency [21], and estimate the quality parameters (brix, texture, internal damages) of fruits in a non-destructive way [22,23].

In real-time sprayer systems, these devices are embedded in conventional tractors and are connected to a global navigation satellite system (GNSS) and computer system that control selectively foliar spray applications [8].

Other systems based on real-time analysis were developed in order to deliver site-specific herbicide applications in arable crops. Besides the multispectral images and the vegetation indices, these systems use a bicameral system, binarization techniques, and morphological algorithms to differentiate between the crop and spontaneous plant species [24,25,26,27].

Both types of real-time precision spraying systems are commercially available or are in process of being commercialized, and have shown a positive reduction in the use of chemical compounds with a reduction of costs and an increase in yield levels (in the case of precision fertilization) compared to traditional sprayers [8].

Image processing techniques with multispectral cameras from visible to near-infrared spectrum are also being used to provide non-destructive plant phenotype image datasets. These approaches have allowed more precise and real-time, high throughput, and high-resolution data for the modelling and prediction of plant growth and morphological development in different conditions, with recent applications in plant health analysis [28,29,30].

More advanced prototypes were made in order to insert these types of sensors in autonomous vehicles. Several terrestrial robotic platforms were developed using multispectral cameras seeking for a more automatic, low-energy cost and accurate analysis of the crop parameters compared to conventional equipped tractors and unnamed aerial vehicles [11,31,32].

Some of these autonomous agricultural devices are capable of identifying weeds and control them using low-dose spraying, mechanical control or thermal control [33]. Other platforms can spray fertilizers in arable crops without the need for heavy tractors, reducing the compaction of the soil and the physical damage to the crops [29]. More recently, modular agricultural robots were developed for mapping different aspects of the cereal plants in parcels of breeding trials [31].

**Figure 1:**
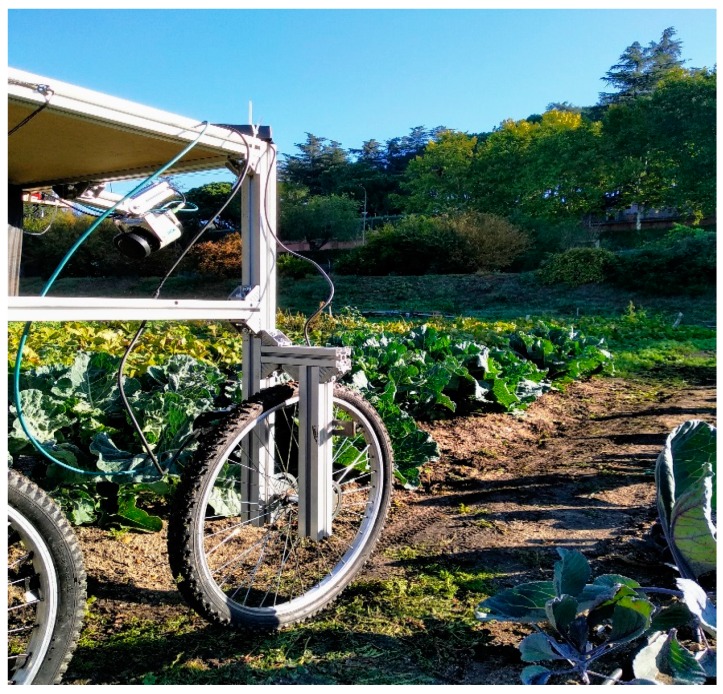
Robotic prototype developed by the SUREVEG team.

Besides the advances in the robotic agricultural platforms, most of them were developed for conventional orchards and arable crops, presenting a lack of technologies in the context of organic horticulture. For this reason, the SUREVEG team has developed the robotic prototype shown in Figure 1.

This prototype is a cart with a manipulator robot and multiple sensors and actuators. The proposed sensors are laser scanners to build three dimensional models of the plants, which will be used to study their growth, and a multispectral camera, which will be used to study the state and health of the crops. Moreover, a system to apply treatments to the soil will be installed in the prototype, consisting of a tank to store the liquid treatment and a sprayer to apply it. Finally, the manipulator robot will be available to accurately place sensors and actuators at the target points.

Having in mind this long-term goal of automatically monitoring crops and applying treatments, this work is focused on the collection of information about the plants by using multispectral imagery. Specifically, the paper describes the acquisition of multispectral images of tomato plants with different levels of organic fertilization, as well as the estimation of their nutritional states in the early stages through multiple vegetation indices and morphological features by using computer vision techniques.

## 2. Materials and Methods

### 2.1. Location and Growing Conditions

The experiment was carried out on a greenhouse in order to obtain the different spectral responses of plants. This greenhouse is in the facilities of the School of Agronomic, Food and Biosystems Engineering of the Technical University of Madrid, which are located in Madrid, Spain (40°26′19.9″ N 3°44′15.7″ W). The tomato (Mina F1 cv.) seedlings were initially cultivated in 3-l pots with height and diameter of 15 cm, filled with a mix of peat substrate (50%) and coconut fiber (50%). After 5 weeks of experiment, the plants were transplanted to 8-l pots with the same mixture.

### 2.2. Trial Design and Fertilization

**Figure 2:**
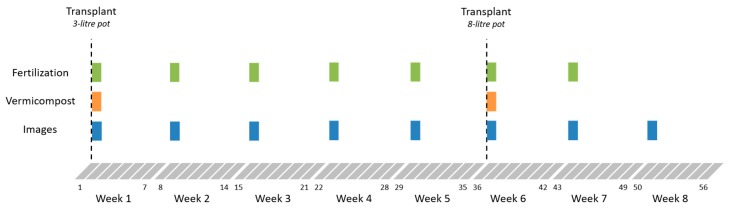
Chronology of plant treatment and image acquisition during the experiment.

The experiment was designed as randomized blocks, with four treatments to be applied to four groups of plants (T0, T1, T2 and T3). The trial design is described by Figure 2, which shows the specific moments when the treatments were applied, and the multispectral images were acquired. The fertilizations were carried out weekly using water-soluble organic fertilizer (3% of Nitrogen and 5% of potassium) obtained from beet vinasse and phosphorite, and registered for use in organic production (COMPO-*Fertilizante Huerto y Frutales*). The initial volume of irrigation and fertilization was 300 mL of solution. One week after transplant, plants were assigned to the different treatments according to the fertilizer label (T0: 0 mL, T1: 6.25 mL, T2: 12.5 mL and T3: 25 mL, which corresponds to T0: 0 g of N and 0 g of K, T1: 0.15 g of N and 0.30 g of K, T2: 0.30 g of N and 0.60 g of K, T3: 0.60 g of N, 1.20 g of K). The plants also received two supplements of vermicomposting: one in the first transplant (T0: 0 mL; T1: 75 mL; T2: 150 mL; T3: 300 mL) and another after the second transplant (T0: 0 mL; T1: 237.5 mL; T2: 475 mL; T3: 950 mL). Images of the plants were acquired with 7-days intervals and processed to compute vegetation indices and analyze them.

### 2.3. System Overview

**Figure 3:**
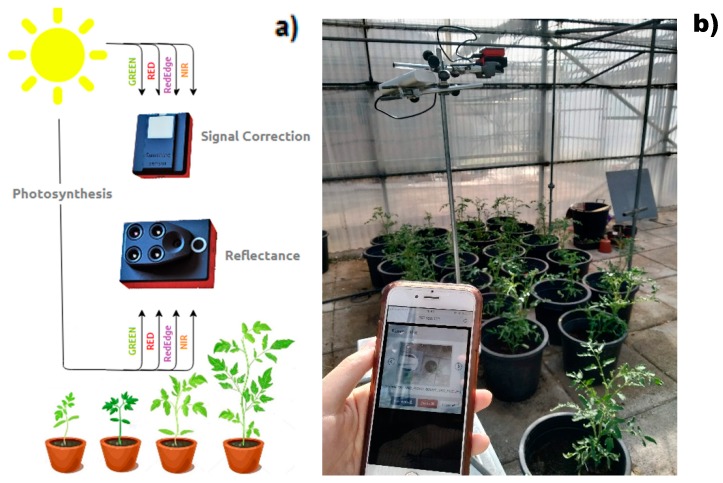
Overview of the image acquisition system. (**a**) Conceptual image acquisition scheme. (**b**) Bracket support with camera and signal correction device measuring tomato plants in early stages.

The general system was composed of images acquired from the greenhouse experiment with different levels of fertilization. The evaluation system was composed of a bracket support stands clamp supporting the sensor and the power supply (battery). The platform has the possibility of height adjustment for the sensor. The distance between the sensor and the bottom of the pot was set at 0.7 m during the first 5 weeks and moved to 1.4 m after Week 5 (Figure 3), since the plants were too high to be observed from the initial distance. The higher distance between the camera and the plant makes the plant image smaller, so the leaf area represented by the pixel area became smaller after that modification. In order to model the data, regression analysis using the mean values was performed, so the results could be extrapolated for the next weeks.

The camera used for image acquisition was a multispectral camera (model Sequoia, Parrot Drones, Paris, France, 2017) which was originally designed for use in agricultural Unmanned Aerial Vehicles (UAV’s). Its internal sensor is composed of four spectral bands which register the reflected light coming from the vegetation and can be used to distinguish plant vigor based on reflectance levels [34]. These bands are: green (550 nm wavelength, 40 nm bandwidth), red (660 nm wavelength, 40 nm bandwidth), red edge (735 nm wavelength, 10 nm bandwidth) and near infrared (790 nm wavelength, 40 nm bandwidth). Additionally, a 16-megapixel RGB camera is also fitted into the commercial system.

The Sequoia Camera has also a sunshine sensor that is used to calibrate the images depending on the sunlight. This makes it possible to compare photos over time, despite variations in light during photo shoots. The sunshine sensor is attached on the upper part to the system, facing the sky and correcting the signal and it also contains a GPS/GNSS module, a magnetometer and inertial measurement system (Figure 3).

The images were taken with and without a black background placed to help the segmentation. The developed algorithms showed different efficiency in segmentation with or without the background. The pots were tagged with a coloured stamp in order to take the images in the same position during the experiment (Figure 3b). The sensor communicates with a smartphone or computer via Wifi protocol in order to store the acquired images.

### 2.4. Computational System for Image Analysis

The computational system used for the image analysis is composed of four major steps: pre-processing, calculation of vegetation indices, image segmentation, and morphological analysis.

#### 2.4.1. Image Pre-Processing

The Sequoia sensor produces tagged image file format (TIFF) images with a size of 1280 × 960 pixels. The camera was designed to take images from a minimum distance of 30 m to the target [26]. When the images were taken with a shorter distance a displacement occurs between the 4-channel images due to an unexpected geometry between the sensors. In order to correct the displacement of the images, an algorithm that shifts an image by a specified number of pixels in either the *x*- or *y*-direction (or both) was used.

The parameters used to define the number of pixels and the direction that each image should be shifted were obtained using a sample image with a referential point and then applied subsequently to all collected data. The red image was arbitrarily selected as the reference, and then the green, near-infrared (NIR) and red-edge images were shifted in order to be in the same position as the red image. The parameters used for the distances (0.7–1.4 m) are shown in Table 1.

**Figure 4:**
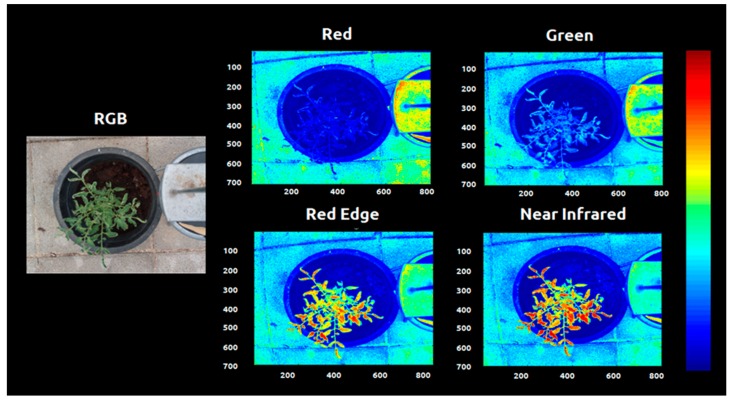
Types of images produced by the sensor after shifting and clipping the area of interest (700 × 800 resolution). (Bluish colors indicate regions with lower levels of reflectance and reddish colors indicates regions with higher levels of reflectance).

The central area of the images was defined as area of interest (AOI) and the consequent processes were used in this area in order to reduce the computational process and optimize the plant recognition and binarization. The images were clipped with AOI-x: 200:900 and AOI-y 200:1000, reducing the size of the image from 960 × 1280 to 700 × 800 squared pixels (Figure 4).

#### 2.4.2. Vegetation Indices

Vegetation indices (VIs) computed from multispectral images are quite simple and effective parameters for the qualitative and quantitative evaluation of vegetation vigour, cover and growth dynamics among other applications. There is a vast implementation of these indices using different airborne and satellite platforms with recent advances using unmanned aerial vehicles (UAV) and tractors. Due to the complexity of the instrumentation platforms, light spectra combinations and resolutions used, there is no unified mathematical expression that attends all applications of VIs [26].

According to the same authors [35], customized algorithms have been studied for several applications combining visible light radiation, mainly the spectral region correspondent with the green region from vegetation, and nonvisible spectra in order to obtain proxy quantifications of the vegetation surface. Therefore, for precise measurement applications, the VIs are optimized and usually constructed according to the specific application requirements, and a validation procedure is needed, along with customized methodologies.

Eleven VIs retrieved from the literature were adopted to find correlations between the fertilization status of the tomato plants and the spectral response. One of the vegetation indices were also used as a filter parameter for binarization of the plant leaves despite the background (image segmentation).

The VIs were chosen based on the literature and in the available bands present at the sensor. The list of indices, abbreviations, formula and traditional application of the VIs can be observed in Table 2. For use in this study, some closest Sequoia reflectance bands were substituted for the traditional narrowband wavelengths.

#### 2.4.3. Plant Extraction (Segmentation)

Digital image processing and computer vision approaches are powerful tools for plant analysis because they allow plant physiological and physical features to be measured non-destructively with high temporal and quantitative resolution [37]. After the image acquisition, the image analysis process starts with the extraction of the numerical data that describes the object in the image. First the background pixels must be separated from the object of interest through a process called object segmentation. The accuracy of this process decreases as the image quality decreases [38]. Multiple plant segmentation methods have been proposed to handle the variation among different sensors including the use of adaptative thresholding and machine learning [39].

**Figure 5:**
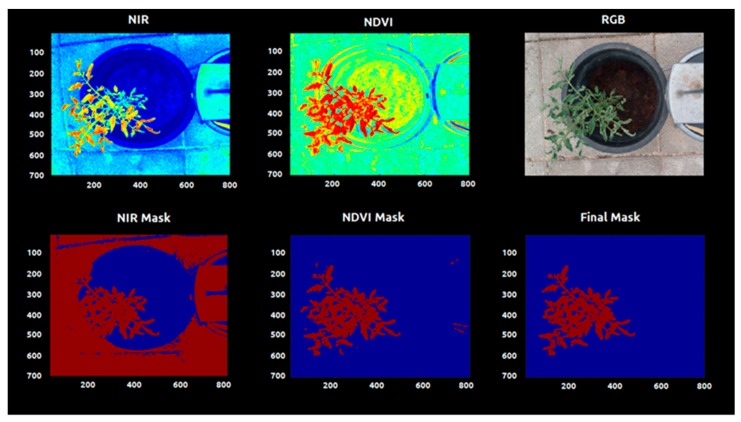
Computer vision process based in NDVI image used to extract the plant from the background. Top images: Near-infrared (left), NDVI (center), and RGB (right); bottom images: binarized images.

Other systems were developed using a fixed threshold and image standardization method [38]. Normalized difference vegetation index (NDVI) has been widely used for distinguishing between plant and soil and plant species through satellite imagery. This occurs because the near infrared (NIR) combined with the red image provides a significantly higher reflectance than soil in natural light conditions [40]. However, NDVI is known for presenting problems related to saturation at extreme values. Aiming to design a robust segmentation algorithm that could be used in different soil conditions, the fixed threshold was avoided. Instead, we used an automatic histogram segmentation that extracted the 92% higher reflectance values from the NDVI images when the NDVI values were superior to 0.1. Another filter based in the NIR image was tested but without reliable results (Figure 5).

**Figure 6:**
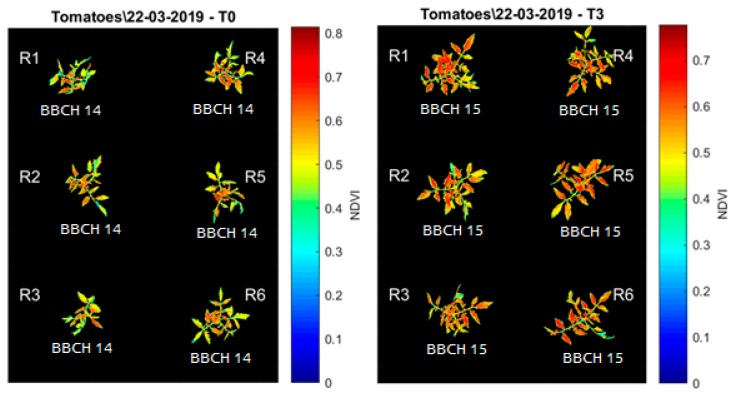
Example of vegetation indices obtained through the Parrot Sequoia camera and segmentation algorithm. NDVI image and phenological state (BBCH scale) of the T0 treatment (left) and the T3 treatment (right).

The images were converted from unit8 to double type in order to allow the pixels to admit decimal values and calculations. All the image processing analysis was performed in MATLAB 2013b (The MathWorks Inc., Natick, MA, USA). After this step, the *bwarefilt* function of MATLAB was applied. This function allows extracting all connected components from the binary image, where the area of the objects is in the specified range producing a new binary image containing only the objects that meet the criterion. In this case, the criterion was to extract the larger object of the image (with more connected components). This allowed the system to eliminate noises and unconnected pixels. The resulting binary image (mask) (Figure 5) was multiplied by the vegetation indices images, in order to extract just the values of VIs present in the plant tissue (Figure 6).

#### 2.4.4. Morphological Analysis

Nutrient deficiency in tomato plants can cause several symptoms besides the change in reflectance of leaves. It is common to observe changes in the plant growing behavior, specially related with morphological aspects of the leaves and shoots.

Nitrogen deficiency can cause restricted shoot growth and small erect leaflets. Phosphorous deficiency can cause restricted shoot growth and small stiffed curved leaves. Deficiency of potassium can cause scorched and curled symptoms in old leaves. Zinc and Iron deficiencies can cause stunting. Boron and Calcium deficiencies can cause changes in the leaflets making them curved and deformed. Copper deficiency symptoms can be observed in the margins of leaflets and younger leaves that curl into a tube shape, the terminal leaves can become very small, stiff and contorted and the stem growth become somewhat stunted [41].

From the binary plant datasets, it is possible to measure plant size, shape, area and other features in an automated way and correlate plant phenotypes with experimental treatments [42,43].

Several other morphological properties can be extracted from binary images using the regionprops function of MATLAB. This function uses the distribution of the pixels to calculate shape parameters and has been recently used for image analysis in medical studies [44], industry [45], plant pathology [46] and plant growth analysis.

These parameters can provide reliable information about the changes in the plants’ area, perimeter, shape and growth behaviour.

Not all parameters resulting from the regionprops function were used, and the description of the morphological properties used for the tomatoes plants analysis can be observed in Table 3.

#### 2.4.5. Statistical Analysis

The experiment used randomized blocks, with four treatments and six repetitions. Each plot was composed of six pots. The statistical analysis carried out through the ANOVA and Tukey’s HSD test with 99% of confidence for average comparisons using the statistical and machine learning toolbox from MATLAB software. ANOVA and Tukey’s HSD test were also performed with 95% confidence, but since there it was no difference in terms of significant factors, the 99% was chosen for this study. For the significant predictor factors, a functional boxplot and a regression analysis (using the week means) was performed in the same software.

## 3. Results and Discussion

Images were collected weekly (one image per plant) during the course of eight weeks. The segmentation of the plant tissue from the background was needed to quantify the spectral response from each plant and to calculate the mean values of the evaluated parameters.

This process also enabled the morphological analysis of the plant growth behavior. Multiple segmentation codes were tested to remove the background, and the code based on the normalized difference vegetation index (NDVI) was the most efficient.

**Figure 7:**
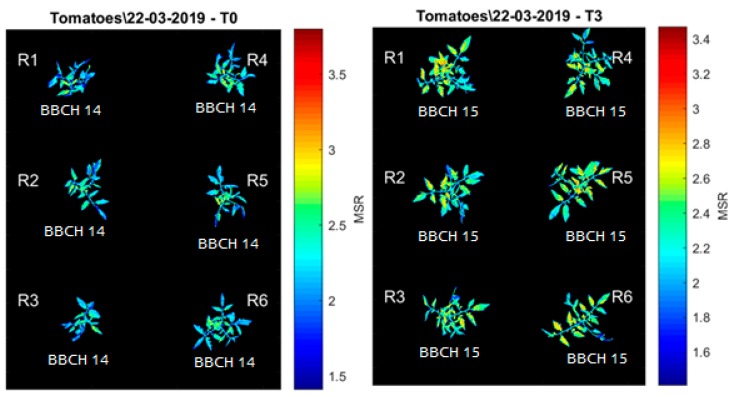
Example of vegetation indices obtained through the Parrot Sequoia camera and segmentation algorithm. Modified Simple Ratio (MSR) image and phenological state (BBCH scale) of the T0 treatment (left) and the T3 treatment (right).

**Figure 8:**
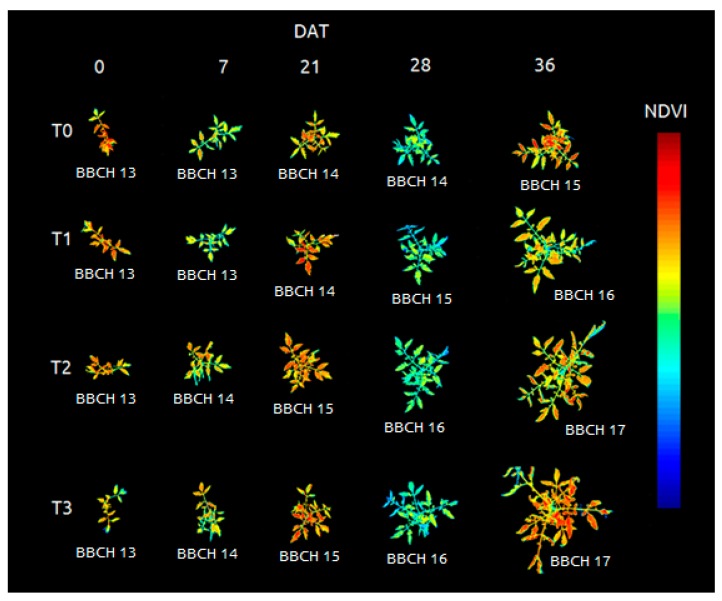
Evolution of the morphology and Normalized Difference Vegetation Index of the tomato plants according to the fertilization treatments, the DAT (days after transplant) and the phenological state (BBCH scale).

All the other bands (red, red edge and green) alone were not able to differentiate the plant tissue from the background. The vegetation indices results were stored by date and treatment in image format seeking a visual confirmation of the segmentation process and a visual representation of the spectral responses (Figure 6, Figure 7 and Figure 8).

The images were taken in the same position during the weeks in order to observe the growth behavior of the plants in the different treatments. Both aspects could be observed during this time: morphological traits and vegetation indices (Figure 8).

The average values of the different vegetation indices per plant were calculated and compared during each week of the experiment. Parameters that showed significant difference and correspondent *p*-values are displayed below (Table 4).

Aiming to identify the most reliable parameters to predict the fertilization level of organic tomato, boxplots were created with the whole amount of data, without week distinction. After that, a comparison test (Tukey’s HSD) was performed to find which parameters are more robust to use in an automatic and nondestructive fertilization level analysis model.

**Figure 9:**
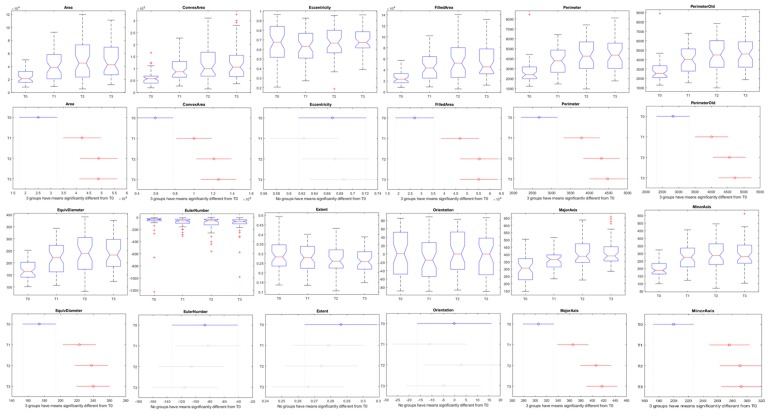
Boxplots and Tukey Test with 99% of probability using all data (50 days) representing morphological parameters of tomato plants with different levels of organic fertilization. T0, T1, T2, and T3 were the four groups of plants treated with different amounts of fertilizer and vermicompost (see Section 2).

**Figure 10:**
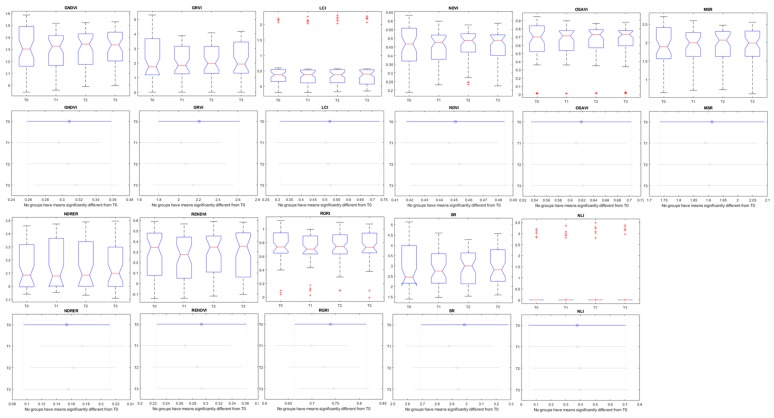
Boxplots and Tukey Test with 99% of probability using all data (50 days) representing vegetation indices of tomato plants with different levels of organic fertilization. T0, T1, T2, and T3 were the four groups of plants treated with different amounts of fertilizer and vermicompost (see Section 2).

The graphical representation of the boxplots and Tukey’s comparison test can be seen in Figure 9. In the case of the morphological aspects, some of the parameters presented significant results with distinction between the control (T0) and the treatments (T1, T2, and T3) (Figure 9). On the other hand, none of the vegetation indices presented a significative difference (with a 99% or 95% level of confidence) to predict the nutritional level of organic tomato plants independent of the week after transplant (Figure 10).

The results presented in Table 4, as well as Figure 9 and Figure 10 indicate that the morphological parameters are more related to the fertilization level of tomato plants at early stages than the spectral responses. These parameters were selected to create regression models in order to predict the nutritional status of the tomato crop.

The regression models were created using the MATLAB function curvefit, the mean values of the control (T0), and the recommended dose (T3), for the first five weeks and excluding outliers. The selected parameters were: area, filled area, convex area, perimeter, equivalent diameter, major axis length and minor axis length (Table 5).

**Figure 11:**
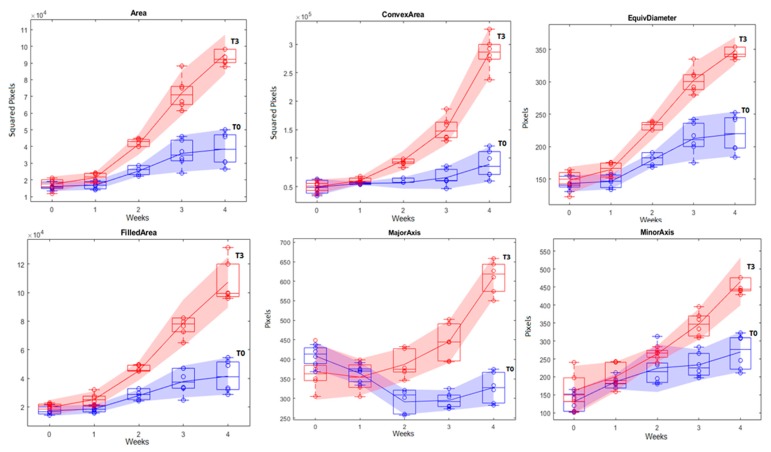
Functional boxplots showing morphological responses extracted from multispectral images correlating with number of weeks after transplant and fertilization treatments with a confidence interval of 95%. T0, T1, T2, and T3 were the four groups of plants treated with different amounts of fertilizer and vermicompost (see Section 2).

Seeking for a more robust, simple and reliable prediction model, functional boxplots were created using the mean values of each plant T0 and T3 for the first four weeks and plotted together. The functional boxplots represent the mean data of each week without the outliers with an interval of confidence of 95% (Figure 11).

### Discussion

The system used for image acquisition showed to be effective for tomato plants at early stages. The same system could be adjusted for tomato plants at late stages, as well as for other plant species and seedlings in different stages (depending on the height adjustments). The pre-processing technique showed to be adequate for the overlay of the Parrot Sequoia images in the different heights tested 0.70 m and 1.40 m.

Plant segmentation is the cornerstone of the image analysis at plant level. Diverse authors used multispectral images to access the nutritional level of the plants. These works are usually focused in small zones or areas, or in multiple plants in a parcel as the minimum management unit. In order to perform precise fertilization at the plant level, the segmentation and extraction of the plant of interest must be also precise. The algorithm developed using the normalized difference index was able to distinguish plant tissue from soil, pot, floor and metal platform extracting the whole plant apart of the background (Figure 4, Figure 5 and Figure 6). The use of NDVI for vegetation extraction and monitoring is widely used for satellite imagery [47,48,49] and this study proves that can also be used with high-resolution plant images. The code was applied in different conditions with good efficacy in extract the plant. The visual inspection was needed to guarantee that the plants were well-segmented and also provides the distribution of the vegetation indices intensity through the leaf tissue. The segmentation also allows morphological analysis during the time, and this system can be useful for digital phenotyping projects. However, the code can present limitations for morphological analysis of plants with presence of senescent leaves since the reflectance of yellow and/or brown leaves are different from the active vegetative tissue.

The vegetation indices used in this study did not show significant differences between the treatments during the initial stages of the plant development, but there were significant differences in the 6th week after the transplant between T0 and T2 and T0 and T1. Among all the studied vegetation indices, the NDVI, GRVI, OSAVI, SR, and MSR were sensible to these differences. Different results were found by [50], that using the crop circle ACS 470 and the SPAD 502 sensors concluded that the vegetation indices based on the red band (NDVI and red vegetation Index) were the best predictors for nitrogen levels in tomatoes maintaining the relationships during the crop.

According to the authors [50], the SPAD readings, the GNDVI, and the GVI were good predictors of the nitrogen status of the crop in the beginning of the cycle, with low accuracy in the later part of the crop cycle. Using the same sensor (Circle ACS 470) and guided sampling method, [51] developed a yield prediction map of tomato crop. Their study used the NDVI index as a predictor parameter and the correlation between the vegetation index and the yield were of 0.67 and 0.71 in two different regions of Spain. Another study conducted by [52] showed the possibility to use vegetation indices to improve the use of nitrogen fertilization in tomatoes cultivated in protected environment. Applying the spectral sensors ASD Field Spec HandHeld 2, and the Minolta SPAD 502, the authors compared the spectral response of tomato plants in the reference plot and with lower levels of nitrogen fertilization.

The use of NDVI and the SPAD measurements as a nitrogen level parameter allowed to reduce significantly the use of nitrogen fertilization maintaining the same yield levels and fruit quality compared to the reference plot [52]. On the other hand, during three years of field trials with different levels of nitrogen fertilization [53] evaluated vegetation indices of tomato plots through the use of a multispectral radiometer (MSR-87, Cropscan Inc., Rochesters, MA, USA). In their study [53], sixteen vegetation indices were analyzed and the NDVI did not show a strong correlation with nitrogen concentration on leaves. It was concluded that the best vegetation index for nitrogen evaluation in tomato was the relation between NIR and the reflectance present in the range of 560 nm (NIR/R560). Some factors can be responsible for the divergence observed between literature results.

The first factor is the type of fertilizer, in all the previous studies, mineral nitrogen fertilization was used. This type of fertilizer is more soluble and less stable and can be absorbed and translocated faster than the organic amendments. The crop stage during the measurements can also be a factor. In the previous studies, the measurements started after the 29th day after transplant where the plants were developed more, and the deficiencies symptoms were more present. The type of sensor is also an important factor because this was the first study to use the Parrot Sequoia sensor at plant level to monitor nutrient deficiency in tomatoes. [54], showed the differences in measurements in different commercial spectral sensors for nitrogen management, and among the studied factors was concluded that the measuring distance, the device temperature and the light intensity can influence the performance of the sensors.

The most interesting results were obtained through the analysis of the morphological traits of the tomato plants. Seven of the twelve aspects analyzed showed significant difference between the control and the fertilized treatments. Only three weeks after the transplant, the plants started to exhibit a significant difference in the plant area, convex area, filled area, minor axis length and equivalent diameter discriminating T3 and T2 of T1 and the control. In the same period, the perimeter was significantly different between the recommended dose T3 and the control, without difference between T2 and T1. The length of the major axis could discriminate between the control against the treatments but not between the treatments already in the third week. In the following weeks, from the fourth week to the end of the study, all the seven morphological traits (area, convex area, filled area, equivalent diameter, perimeter, minor axis length and minor axis length) showed significant differences between the control and the treatments, except for the major axis length in the seventh week after transplant.

These results are in agreement with the results described in the literature. Mooy et al. [55] showed the positive influence of soluble organic fertilizer in tomato plant height and the number of leaves. Other studies also demonstrated that the nitrogen and phosphorous fertilization influences the fresh shoot weight, plant height, stem diameter, leaf number, and leaf area of tomato seedlings [56,57,58,59]. In field trials, the nitrogen fertilization produced an increase in the leaf area index and in the above-ground weight of tomato plants [60] and the use of NPK induce an increase in plant height and in the number of leaves [61]. Besides the slight difference in the morphological parameters between the fertilized treatments T1, T2, and T3, there was no significant difference with 1% of probability, because, for the functional boxplot analysis, the treatment with the recommended dose (T3) was chosen for comparison with the control (T0).

The use of functional boxplots as a predicting model is interesting because they can exclude outliers and can be used as a threshold parameter [62]. As can be observed, the functional boxplots displays the range of the observed values until the 4th week after transplant and not until the Week 7. At Week 5 the plants were too high to be observed with 0.70 m height as was planned for the robotic prototype, so the camera bracket was set for a 1.4 m height. This adjustment in the camera distance cause differences in the relationship between morphological parameters and the number of pixels.

## 4. Conclusions

The computer vision methodology developed in this article was suitable for monitoring the development of tomato var. Mina F1 at early stages with different levels of organic fertilization in a protected environment. The methodology involves consecutive steps, and using an automatic and non-destructive approach provides several morphological and spectral aspects of the plants that can be used in a robotic platform.

After the pre-processing, the NDVI threshold showed to be crucial and effective for plant extraction. Among the several spectral (vegetation indices) responses observed, the NDVI, OSAVI, simple ratio, GRVI, and modified simple ratio were the only ones that showed a small difference between treatments in the sixth and seventh weeks after transplant, but without practical applications.

Studying the evolution of a crop by using multiples images allowed to extract several morphological features. The automatic morphological analysis was able to distinguish between the control and the fertilized treatments from the third week after transplant until the end of the experiment with 99% of confidence according to the Tukey’s honest significant differences test.

Among the studied morphological parameters, area, convex area, filled area, perimeter, major axis length, minor axis length and equivalent diameter showed to be useful to distinguish between the control and fertilized treatments. Convex area was the parameter that presented the highest difference between control and the fertilized plants according to the functional boxplot analysis.

This was the first study to relate the use of morphological features extracted automatically with the organic fertilization level in vegetable plants. The image analysis system was able to distinguish plant tissue from soil, floor, and metal structures. Therefore, most of the image processing steps can be extrapolated to other vegetable cultures, although future analysis is necessary to evaluate its robustness. Moreover, the Parrot Sequoia camera fulfilled the requirements to be used in the robotic platform and besides be originally developed for aerial images this study proved the capacity of its use at short distances.

Additionally, the same methodology can be used for analyzing other aspects related with morphology features in tomato plants, such as disease severity, hydric and salinity stress or any environmental modification that can cause aboveground changes in the morphology or spectral responses of vegetable plants. This technique has potential to be used in many branches of plant health studies and provides an innovative and automatic way to measure the severity of abiotic disorders.

In a subsequent investigation, we are developing an extended version of the methodology to be applied using the robotic platform at the field level. Trials are being conducted in an experimental line with different vegetable crops to study the best image acquisition interval in a scenario with movement. Other trials using the robotic platform are being conducted in an experimental field area with different crops such as onion, pumpkins, tomatoes, and zucchini with different levels of fertilization. In these trials, the multispectral camera is being integrated with laser sensors (Lidar) for a multiscale analysis of the crops.

## Figures and Tables

**Table 1:** Shift factors (*x*- and *y*-direction) used for overlaying the different images using parrot sequoia in shorter distance of plant samples. (Red image used as reference).

Images	Shift Factor
Green	[50, 23]
Near Infrared	[41, −34]
Red Edge	[68, −11]

**Table 2:** Vegetation Indices (VIs) used to create spectral profiles of the tomato plants from the multispectral data with formulae and traditional applications (obtained from [36]).

Index	Abbreviation	Formula	Application
Normalized Difference Vegetation Index	NDVI	$\frac{\rho \mathrm{NIR}\;–\;\rho \mathrm{RED}}{\rho \mathrm{NIR}+\;\rho \mathrm{RED}}$	Measuring green vegetation through normalized ration ranging from −1 to 1.
Index	GNDVI	$\frac{\rho \mathrm{NIR}\;-\;\rho \mathrm{GREEN}}{\rho \mathrm{NIR}+\;\rho \mathrm{GREEN}}$	Modification of NDVI, more sensitive to chlorophyll content.
Normalized Difference Vegetation Index	RENDVI	$\frac{\rho \mathrm{NIR}\;-\;\rho \mathrm{RedEdge}}{\rho \mathrm{NIR}+\;\rho \mathrm{RedEdge}}$	Modification of NDVI, using Red-Edge information related to plant health.
Green Normalized Difference Vegetation Index	NLI	$\frac{{\rho \mathrm{NIR}}^{2}\;-\;\rho \mathrm{RED}}{{\rho \mathrm{NIR}}^{2}+\;\rho \mathrm{RedEdge}}$	Modification of NDVI used to emphasize linear relations with vegetation parameters.
Red-Edge Normalized Difference Vegetation Index	OSAVI	$1.5*\;\frac{\rho \mathrm{NIR}\;-\;\rho \mathrm{RED}}{\rho \mathrm{NIR}+\;\rho \mathrm{RED}+0.16}$	Variation of NDVI in order to reduce the soil effect
Nonlinear Vegetation Index	GRVI	$\frac{\rho \mathrm{NIR}\;}{\;\rho \mathrm{GREEN}}$	Related with leaf production and stress
Optimized Soil Adjusted Vegetation Index	MSR	$\frac{\left(\frac{\rho \mathrm{NIR}\;}{\;\rho \mathrm{RED}}\right)-1\;}{\sqrt{\left(\;\frac{\rho \mathrm{NIR}\;}{\;\rho \mathrm{RED}}\;\right)}+1}$	A combination of renormalized NDVI and SR to improve sensitivity to vegetable characteristics
Green Ratio Vegetation Index	SR	$\frac{\rho \mathrm{NIR}\;}{\rho \mathrm{RED}}$	Ratio of NIR scattering to chlorophyll and light absorption used for simple vegetation distinction
Modified Simple Ratio	NDRER	$\frac{\rho \mathrm{RedEdge}\;-\;\rho \mathrm{RED}}{\rho \mathrm{RedEdge}+\;\rho \mathrm{Red}}$	Modification of NDVI, using Red-Edge instead of NIR.
Simple ratio	SPI2	$\frac{\rho \mathrm{NIR}\;-\;\rho \mathrm{GREEN}}{\rho \mathrm{NIR}-\;\rho \mathrm{RED}}$	Index used in areas with high variability in canopy structure
Normalized Difference Red-Edge/Red	LCI	$\frac{\rho \mathrm{NIR}\;-\;\rho \mathrm{RedEdge}}{\rho \mathrm{NIR}+\;\rho \mathrm{RED}}$	Index to assess chlorophyll content in areas of complete leaf coverage.

Note: For use in this study, some closest Sequoia reflectance bands were substituted by the traditional narrowband wavelengths as described by [36] and showed in the Table 2.

**Table 3:** Morphological properties calculated for the tomato plants at early stages images with different levels of organic fertilization.

Morphological Property	Description
Area	Actual number of pixels in the region, returned as a scalar.
Convex Area	Number of pixels in the image that specifies the convex hull, with all pixels within the hull filled in (set to on), returned as a binary image (logical). The image is the size of the bounding box * of the region.
Eccentricity	Eccentricity of the ellipse that has the same second-moments as the region, returned as a scalar. The eccentricity is the ratio of the distance between the foci of the ellipse and its major axis length. The value is between 0 and 1. (0 and 1 are degenerate cases. An ellipse whose eccentricity is 0 is a circle, while an ellipse whose eccentricity is 1 is a line segment.)
Diameter Equivalent	Diameter of a circle with the same area as the region, returned as a scalar. Computed as $\sqrt{4*\frac{Area}{\pi }}$.
Euler Number	Number of objects in the region minus the number of holes in those objects, returned as a scalar.
Extent	Ratio of pixels in the region to pixels in the total bounding box *, returned as a scalar. Computed as the area divided by the area of the bounding box *.
Filled Area	Number of on pixels in filled image, returned as a scalar.
Orientation	Angle between the *x*-axis and the major axis of the ellipse that has the same second-moments as the region, returned as a scalar. The value is in degrees, ranging from −90 degrees to 90 degrees.
Major Axis Length	Length (in pixels) of the major axis of the ellipse that has the same normalized second central moments as the region, returned as a scalar.
Minor Axis Length	Length (in pixels) of the minor axis of the ellipse that has the same normalized second central moments as the region, returned as a scalar
Perimeter	Distance around the boundary of the region returned as a scalar. This function computes the perimeter by calculating the distance between each adjoining pair of pixels around the border of the region
Solidity	Proportion of the pixels in the convex hull that are also in the region, returned as a scalar. Computed as area/convex area

* Bounding box: Smallest rectangle containing the region.

**Table 4:** *p*-values for the parameters (that presented significative difference) analyzed with multispectral images in tomato plants with different organic fertilization levels. (ANOVA Significance test 99%).

	Time After Transplant (Days)							
	08-mar	15-mar	22-mar	29-mar	05-abr	12-abr	19-abr	26-abr
	0	7	14	21	28	36	43	50
**NDVI**	n.s	n.s	n.s	n.s	n.s	n.s	0.02 **	0.05 **
**MSR**	n.s	n.s	n.s	n.s	n.s	n.s	0.074 **	0.03 **
**GRVI**	n.s	n.s	n.s	n.s	n.s	n.s	0.013 **	0.103 **
**Area**	n.s	n.s	n.s	0.002 **	<0.0001 **	<0.0001 **	<0.0001 **	<0.0001 **
**MajorAxisLength**	n.s	n.s	n.s	<0.0001	<0.0001 **	0.001 **	0.0008 **	<0.0001 **
**MinorAxisLength**	n.s	n.s	n.s	0.0007 **	<0.0001 **	<0.0001 **	<0.0001 **	<0.0001 **
**ConvexArea**	n.s	n.s	n.s	<0.0001 **	<0.0001 **	<0.0001 **	<0.0001 **	<0.0001 **
**FilledArea**	n.s	n.s	n.s	0.02 **	<0.0001 **	<0.0001 **	<0.0001 **	<0.0001 **
**Perimeter**	n.s	n.s	n.s	0.0016 **	<0.0001 **	<0.0001 **	<0.0001 **	0.0002 **
**EquivDiameter**	n.s	n.s	n.s	<0.0001 **	<0.0001 **	<0.0001 **	<0.0001 **	<0.0001 **

**: Significative values with 99% of confidence.

**Table 5:** Linear and polynomial regressions correlating morphological parameters were acquired using multispectral images to weeks after transplant in different organic fertilization levels in tomato plants at early stages.

Parameter	Tr.	Regression	*R* ^2^
Area	T0	f(x)=10070x+26550	0.937
	T3	$f\left(x\right)=2825x^{2}+3078x+8909\;$	0.983
Filled Area	T0	f(x)=6766x+8284	0.953
	T3	$f\left(x\right)=3118x^{2}+2072x+13130\;$	0.977
Perimeter	T0	f(x)=164.5x+2211	0.559
	T3	$f\left(x\right)=381.7x^{2}-\;1065x+3265$	0.941
Eq. Diameter	T0	f(x)=21.93x+114.4	0.938
	T3	f(x)=52.65x+80.43	0.971
Convex Area	T0	$f\left(x\right)=2984x^{2}-\;8845x+56880$	0.957
	T3	$f\left(x\right)=19530x^{2}-60980x+95950$	0.992
Major Axes	T0	$f\left(x\right)=16.53x^{2}-112.2x+522.2\;$	0.933
	T3	$f\left(x\right)=27.41x^{2}-106.9x+452.5$	0.988
Minor Axes	T0	$f\left(x\right)=-4.833x^{2}+61.4x+77.95$	0.976
	T3	f(x)=71.54x+69.48	0.964
